# Influence of Metallic Powder Characteristics on Extruded Feedstock Performance for Indirect Additive Manufacturing

**DOI:** 10.3390/ma14237136

**Published:** 2021-11-24

**Authors:** Cyril Santos, Daniel Gatões, Fábio Cerejo, Maria Teresa Vieira

**Affiliations:** 1 CDRSP—Centre for Rapid and Sustainable Product Development, Polytechnic Institute of Leiria, Rua General Norton de Matos, Apartado 4133, 2411-901 Leiria, Portugal; 2 CEMMPRE—Centre for Mechanical Engineering, Materials and Processes, University of Coimbra, Pinhal de Marrocos, 3030-788 Coimbra, Portugal; daniel.gatoes@uc.pt (D.G.); TERESA.VIEIRA@dem.uc.pt (M.T.V.); 3 IPN—Pedro Nunes Institute, Rua Pedro Nunes, 3030-199 Coimbra, Portugal; phcerejo@gmail.com

**Keywords:** additive manufacturing, copper, feedstock, MEX, filament, micro-CT

## Abstract

Material extrusion (MEX) of metallic powder-based filaments has shown great potential as an additive manufacturing (AM) technology. MEX provides an easy solution as an alternative to direct additive manufacturing technologies (e.g., Selective Laser Melting, Electron Beam Melting, Direct Energy Deposition) for problematic metallic powders such as copper, essential due to its reflectivity and thermal conductivity. MEX, an indirect AM technology, consists of five steps—optimisation of mixing of metal powder, binder, and additives (feedstock); filament production; shaping from strands; debinding; sintering. The great challenge in MEX is, undoubtedly, filament manufacturing for optimal green density, and consequently the best sintered properties. The filament, to be extrudable, must accomplish at optimal powder volume concentration (CPVC) with good rheological performance, flexibility, and stiffness. In this study, a feedstock composition (similar binder, additives, and CPVC; 61 vol. %) of copper powder with three different particle powder characteristics was selected in order to highlight their role in the final product. The quality of the filaments, strands, and 3D objects was analysed by micro-CT, highlighting the influence of the different powder characteristics on the homogeneity and defects of the greens; sintered quality was also analysed regarding microstructure and hardness. The filament based on particles powder with D_50_ close to 11 µm, and straight distribution of particles size showed the best homogeneity and the lowest defects.

## 1. Introduction

**Figure 1:**
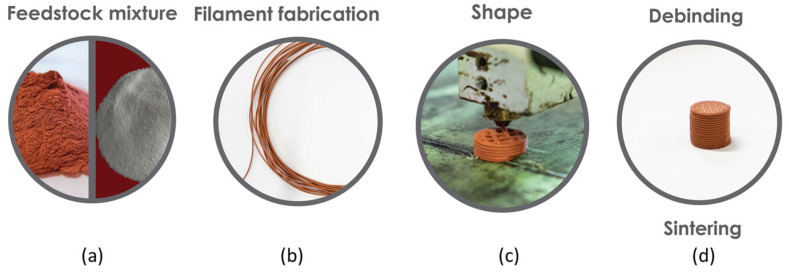
Stages of MEX for building 3D objects: (**a**) feedstock; (**b**) filament; (**c**) shaping; (**d**) debinding and sintering.

Additive manufacturing (AM) has gained a great amount of interest in the past two decennia for various fields of applications [1]. After the boom of direct processes (i.e., selective laser melting (SLM), electron beam manufacturing (EBM), direct energy deposition (DED)), two indirect technologies assume more and more the future (binder Jetting (BJ) and material extrusion (MEX)), due to their simplicity, reliability, low cost (i.e., equipment) and a wide range of different printing materials available. The latter is well known, particularly for polymeric materials, under the name of fused deposition modeling (FDM). When applied to the mixing of metallic/ceramic powder particles and organic binder and/or additives based on polymers, it has adopted the standardised name of MEX [2,3,4,5,6]. This process for 3D object shaping is based on FDM, but filament manufacturing is similar to powder metal extrusion process (PEP) and powder injection moulding (PIM). Both these processes use, as feedstock, polymers and organic materials with the highest feasible metal powder content, designated by critical powder volume concentration (CPVC) [7]. A feedstock must consist of a powder with optimal characteristics and an appropriate binder, which are determinant factors to achieve quality in the final 3D object [8,9]. Similarly to PEP/PIM, the parts produced by MEX use filaments with a high volume percentage of metals particles, typically between 50 and 65 vol.% [10,11,12], and need two subsequent steps: debinding, to promote binder removal, and sintering, to attain a dense 3D object (Figure 1). Nevertheless, PIM feedstocks result in a brittle filament form; thus, it is necessary to optimise the mixture to achieve filament requirements for MEX, such as a good balance between suitable rheology properties, stiffness, and flexibility.

Nowadays, commercial filaments with metal particles are emerging, although most of them have a low metal powder content or use PLA or ABS as the binder, which means that the main goals are the aesthetic appearance of the products or to increase properties of the master polymer [13,14].

For stainless steel, different studies reveal that powder must have (3Ss): particle size D50 from 5 µm to 15 µm, a monomodal particle size distribution, and a shape factor close to 1 [12], similar to the ones used in PIM.

Copper, particularly pure copper, is a widely applied metal in engineering applications due to the combination of suitable mechanical strength and excellent thermal and electrical properties (391 W/(m·°C) and 103.6% IACS, respectively, at 20 °C) [15]. However, considerable research studies and advancements are still needed in processing copper by AM to match the industry requirements and process repeatability. A few studies investigated copper using SLM [2,16]. Although SLM has been demonstrated to successfully work with a comprehensive range of metals, it has disadvantages when it comes to the fabrication of copper due to the high reflectivity of this metal to the laser beam, particularly for near-infrared radiation [17], and high thermal conductivity that leads to rapid heat dissipation and an uncontrolled molten pool [18], thus resulting in parts with significant porosity (11.9–17.0%) [19,20,21]. In order to achieve dense copper parts, a higher laser energy density is required, which means a higher power laser source and, therefore, an increase in energy consumption. Some studies regarding SLM of pure copper using a greater output laser power reported theoretical density values between 96.6 and 99.4% in self-developed platforms [22,23,24]. These are the reasons for the selection of copper powder as a material to highlight the role of the MEX as an effective processing technology.

In summary, the production of 3D objects made of copper powder by direct AM, particularly those associated with the liquid state (SLM), entails several problems surmounted with the indirect MEX process. In addition to significant research on the role of powder characteristics, studies focus essentially on steel powder. However, the properties of steel and copper powder oblige a detailed analysis of these powder particles. Furthermore, according to the available literature, no scientific studies have investigated the influence of the application of MEX technology for the production of densified copper parts, in spite of the company Markforged having commercialised copper filaments for MEX recently [25].

According to the state of the art, no studies have investigated the influence of copper powder particles characteristics on the products made by MEX technology. It is, therefore, crucial to have a complete understanding of all five steps involved in the manufacturing process—namely, mixing the constituents of the filaments (copper powder, binder, and additives), extrusion (filament manufacturing), strands for shaping (3D objects), debinding (binder removal), and sintering (powder particle consolidation).

## 2. Characterisation Techniques and Material

### 2.1. Experimental Conditions

The powder particle size and the particle size distribution were measured by laser diffraction spectrometry (LDS) on a Malvern Instruments Mastersizer 3000 (Malvern Instruments Ltd., Worcestershire, UK), following ISO 13320:2009. The analysis of particles powder, filament, and strands morphology was made by scanning electron microscopy (SEM) on a Tescan Vega 3 (Tescan, Brno, Czech Republic) and an FEI Quanta 400FEG (FEI Europe BV, Eindhoven, The Netherlands). The phasic structure of the powder and greens were evaluated by X-ray diffraction (XRD) on a Philips X’Pert diffractometer (Philips, Eindhoven, The Netherlands); according to EN 13925:2003, the current intensity was 35 mA and voltage of 40 kV. The wavelength radiation was cobalt (Kα1 = 0.17810 nm and Kα2 = 0.17928 nm). The acquisition conditions used a Bragg–Brentano (θ–2θ) geometry (40° ≤ 2θ ≤ 80°) and a step of 0.04°/s per point. The thermogravimetric analyses (TGA) were performed on a PerkinElmer STA 6000 (Waltham, MA, USA).

The filaments were analysed by a non-destructive method using X-ray microcomputed tomography (micro-CT), Bruker SkyScan 1275 (Bruker, Kontich, Belgium). An acceleration voltage of 80 kV and a beam current of 125 μA was set using a 1 mm copper filter and step-and-shoot mode. Pixel size was set to the equipment minimum of 5.67 μm, and the random mode was used. In total, 1056 projection images were acquired at 0.2° angular step with 3 frames average per step, using an exposure time of 65 ms. Regarding the strands, they were scanned with an acceleration voltage of 50 kV and a beam current of 80 μA, using as a filter aluminium with 1 mm thickness and an exposure time of 230 ms. The 3D objects and sintered filaments were scanned using an acceleration voltage of 100 kV and a beam current of 100 μA, using a 1 mm copper filter. Then, 3D objects’ projection images (529) were acquired at 0.4° angular step with 8 frames average per step, using an exposure time of 245 ms, and sintered filaments projection images (1056) were acquired at 0.2° angular step with 3 frames average per step and a 225 ms exposure time. All omitted conditions on the strands and parts were similar to the ones used on the filament. The micro-CT images were reconstructed with dedicated manufacturer software.

Hardness measurements were performed with a microhardness tester Shimadzu HMV (Shimadzu Corporation, Kyoto, Japan). For each measurement, a load of 98 mN was applied for 15 s by a Vickers indenter.

### 2.2. Copper Powder Characterisation

Three copper powders with essentially two different characteristics—particle size and particle size distribution—were tested. A similar organic mixing based on polymeric materials (master binder, backbone, and plasticiser) with identical thermal cycle heat treatments for debinding and sintering were mixed (Table 1) [26]. In addition to the selected binder (a mixture of polyolefin waxes and ethylenic polymers) [27], the additives chosen are necessary to attain a feasible stiffness (backbone) and flexibility (plasticiser) in a filament, which means a thermoplastic elastomer (TPE) and a plasticizer, respectively. The green filaments developed based on this procedure were constituted by copper powder (purity 99.99%), with different particle sizes and particle size distributions.

The three different copper powder particles highlighted the role of particle size and particle size distribution of particles in the quality of filament, strands, and consequently, in the final product, and were furnished by Ecka (Ecka Granules GmbH, Fürth, Germany—type A) and Alfa Aesar (Alfa Aesar, Haverhill, MA, USA—type B,C) (Table 2).

**Figure 2:**
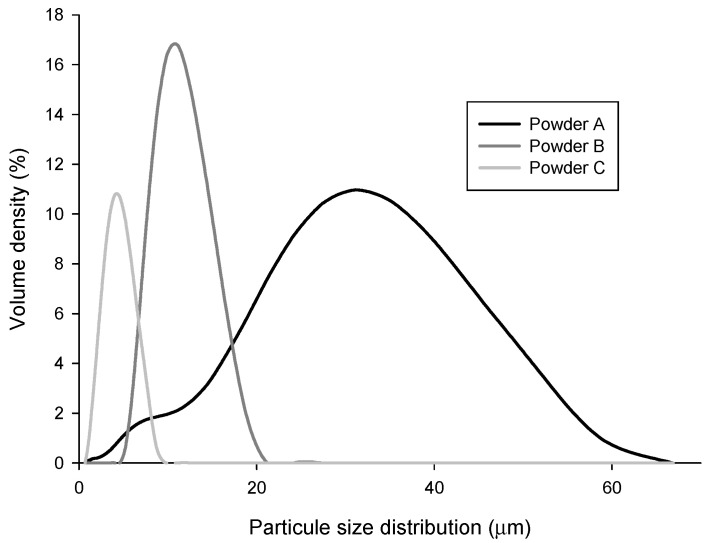
Particle size distribution of the different copper powder particles.

The powder particle sizes were chosen as D_50_ equal to (A) 28.00 μm, (B) 11.30 μm, and (C) 3.97 μm, and particle size distribution ranged from a bimodal with wide distribution (A) up to monomodal with a very narrow distribution (C) (Figure 2).

Table 2 shows the different particle size distribution of the three different copper powders selected in this study.

The density values revealed pre-oxidation of powder particles B and C (density of Cu = 8960 Kg/m^3^; density Cu_2_O = 6310 Kg/m^3^) [28]. Moreover, the colour of the different copper powder particles indicated the presence of Cu_2_O on the powder surface (B,C).

**Figure 3:**
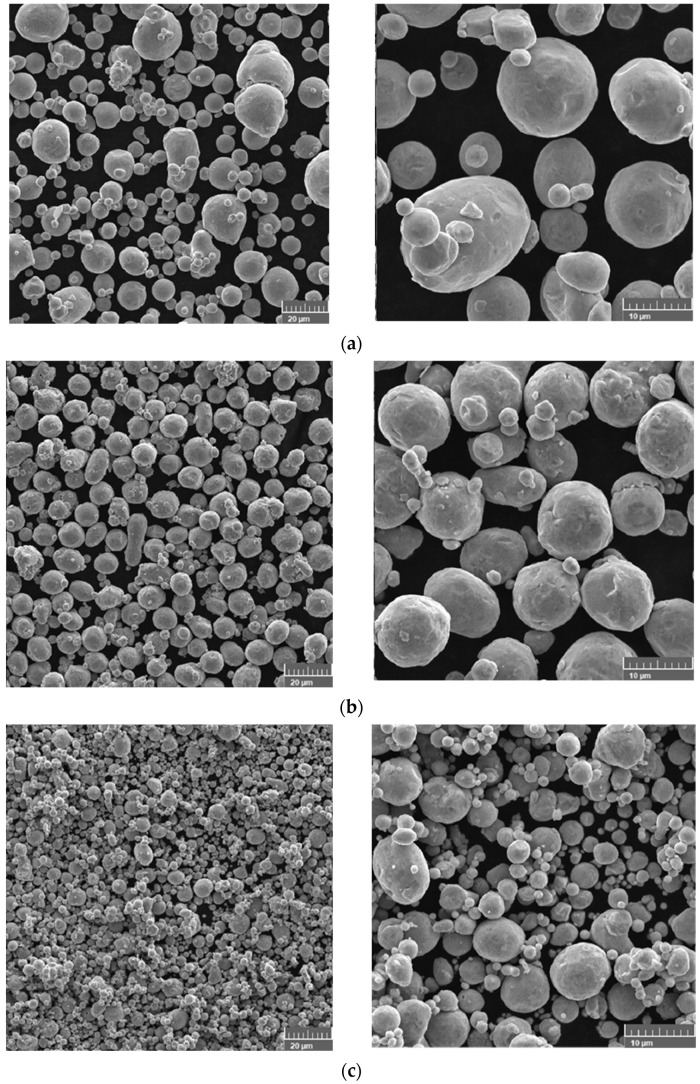
Morphology of powder particles (SEM): (**a**) powder A, (**b**) powder B, and (**c**) powder C.

Regardless of the selected copper powder, the shape factor (D_maximum_:D_minimum_) was close to 1 (Figure 3).

**Figure 4:**
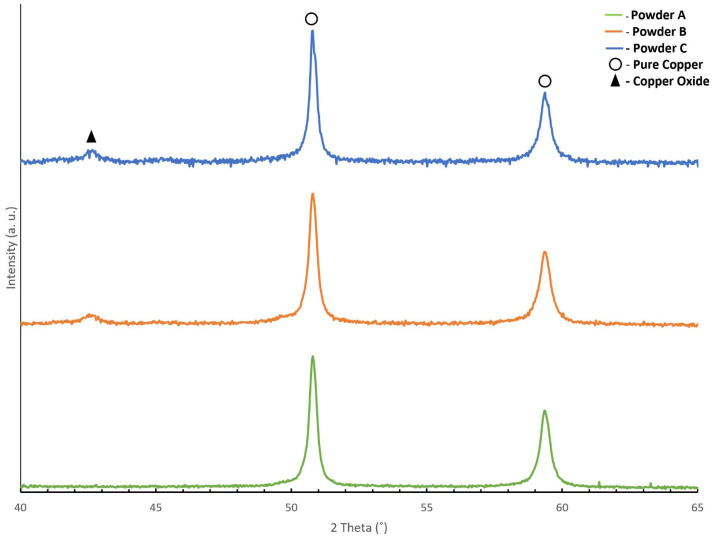
X-ray diffractograms of powder A (**bottom**), powder B (**middle**), and powder C (**top**).

XRD of the highest D_50_ powder showed pure copper (ICDD 04-0836), and the presence of copper oxide phase (ICDD 075-1531) was not distinguishable (Figure 4). However, in powder particles B and C, the XRD diffractogram detected strong peaks of Cu and weak peaks of copper oxide. The weak peak of copper oxide confirmed the slight involvement of Cu with the atmospheric oxygen during conventional atomisation of powder, showing that a small oxide phase was present in the powder surface.

Table 3 summarises the 4Ss (particle Size, particle Size distribution, Shape, and Structure (topography and phases) of the copper powder particles used in this work.

## 3. Experimental Methodology

### 3.1. Processing of Filament Feedstocks

#### 3.1.1. Evaluation of CPVC

The feedstocks were optimised in a torque rheometer (Plastograph^®^ Brabender W 50, Brabender GmbH & Co. KG, Duisburg, Germany) at a temperature of 180 °C, 30 rpm, and a 38.5 cm^3^ mixing chamber. The strategy was to establish the best compromise between the maximum volume concentration of copper powder in the feedstock and its extrudability, which leads to the best conditions to extrude the filament without disruptions. This compromise is named CPVC [27]. The optimum volume ratio has been widely studied in research studies related to PIM. In the processing of filaments (extrusion), the methodology was similar, but the role of backbone and plasticiser needed to manufacture a filament must be highlighted.

#### 3.1.2. Filaments

After the evaluation of CPVC feedstocks, they were granulated into small pellets and extruded in filament form, using a single screw extruder (Brabender GMBH & Co. E 19/25 without calibration system) and a nozzle of 1.75 mm. The temperatures in different zones of the extrusion cylinder were 170, 175, and 180 °C (nozzle).

In order to support the quality of filament for the additive process (MEX), a function of powder characteristics, several tests of tensile and flexural strength were performed. The equipment was a Stable MicroSystems, Godalming, with a 5 kN loading cell; tensile tests were carried out with a velocity of 0.5 mm/min and a gauge length of 10 mm; for the three-point bending test, the loading span was 20 mm, and the cell load velocity of 0.5 mm/min. For both tests (tensile and bending), six specimens (green filament) were tested for each reference powder particle (A, B, and C).

### 3.2. Three-Dimensional (3D) Printing

The filament was extruded in a Prusa i3 MK3S (Prusa Research, Prague, Czech Republic) through a 0.4 mm nozzle diameter. The nozzle temperature was 200 °C, and the platform temperature was 50 °C for the first layer and 80 °C for the remaining layers. The print speed was 30 mm/s. The extrusion multiplier was set to 1.15 to offset the overlap and create a more homogenous layer.

### 3.3. Processing Conditions after Shaping

#### 3.3.1. Debinding

**Figure 5:**
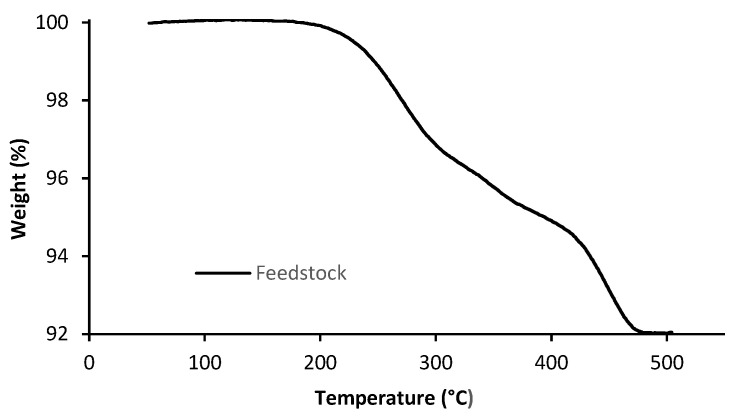
TGA of feedstocks (powder A).

The master binder and additives must be completely removed during the debinding step. The elimination of the polymeric component is critical in the shaping, debinding, and sintering (SDS) process. The type of debinding selected was based on thermal gravimetric analysis (TGA) of the filaments. The TGA curve highlights the temperatures during the heating process where the loss of weight is disruptive, which means the temperatures at which the organic constituents of feedstock are ustulated. The TGA curve represents the weight loss evolution of the feedstock studied with temperature, in an inert atmosphere of Ar + H_2_ (5 vol.% H_2_) and a heating rate of 1 °C/min. From this curve, it became clear that the temperature of 500 °C was enough to eliminate all the binder and additives of the feedstock (7.5 wt.% = 39 vol.%) (Figure 5).

**Figure 6:**
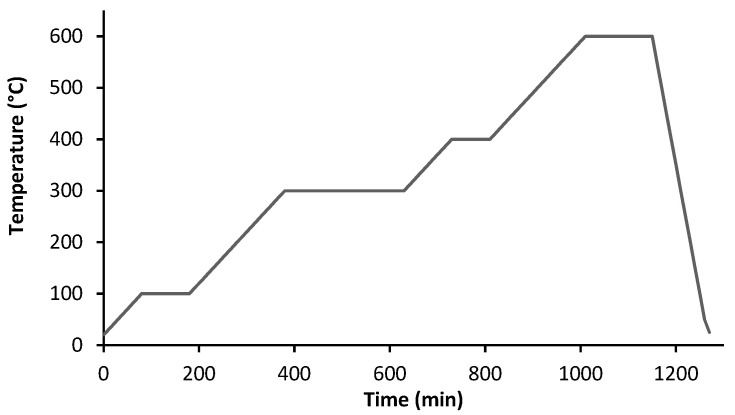
Thermal cycle of debinding.

Figure 6 shows the thermal cycle selected for an efficient debinding of the parts (brown). The conditions were similar to the TGA, with the atmosphere being Ar + H_2_ (5 vol.% H_2_) and a heating rate of 1 °C/min.

#### 3.3.2. Sintering

**Figure 7:**
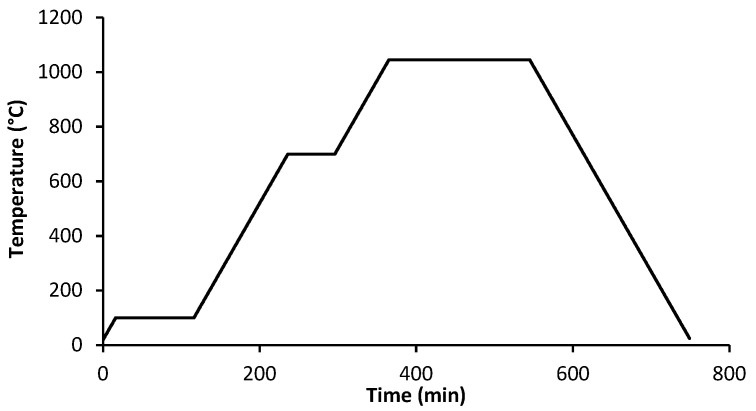
Thermal cycle of sintering.

The brown parts were sintered in the same atmosphere of debinding, but the heating rate was 5 °C/min and the maximum temperature was 1045 °C, for 3 h (Figure 7).

#### 3.3.3. Micrographic Analysis of Green and Sintered Filament

The filament green was analysed without polishing and etching. However, after sintering, the specimens of filament were polished and chemically etched for optical microscopy analysis (Nikon OPTIPHOT metallographic polarising microscope, Tokyo, Japan). For the evaluation of the grain size (ASTM 407) and microstructure, the selected etchant was iron chloride, hydrochloric acid, water, and glycerol (1:1: 3:5), and the duration of etching was 1 min.

## 4. Results and Discussion

### 4.1. Optimisation of Feedstocks for Copper Filaments

For the development of a new copper filament for material extrusion (MEX), several feedstocks were developed using torque rheometry equipment. CPVC established was 61 vol.% [29]. Table 4 shows the final composition and density of each feedstock, with its associated torque.

### 4.2. Green

#### 4.2.1. Filaments

The extruded filaments, all produced under the same conditions, had variations in diameter depending on the dimension and distribution of copper particles. Thus, filaments had the following diameters: for powder A = 1.76 mm, B = 1.70 mm, and C = 1.68 mm. The variation in the filaments was not significant, i.e., negligible.

**Figure 8:**
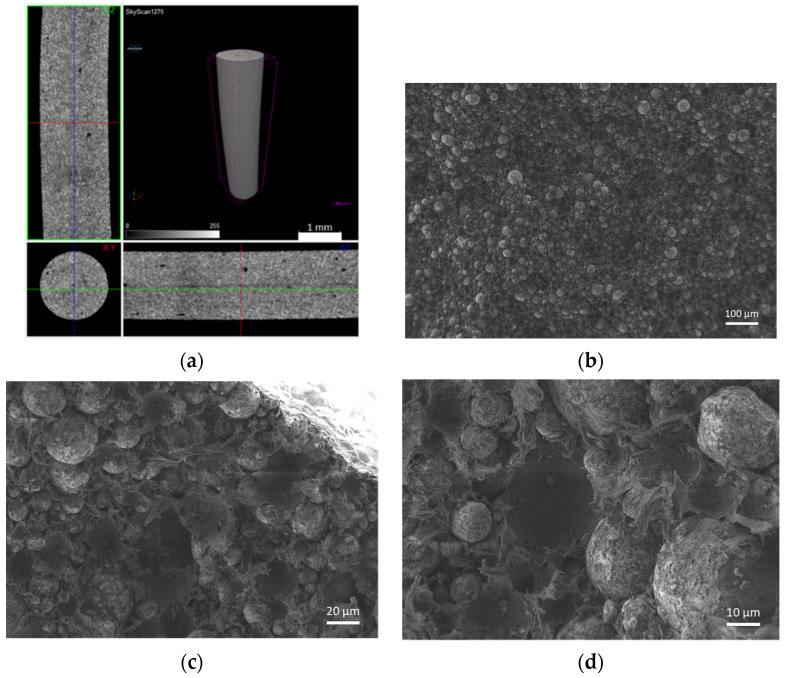
Filament A (green): (**a**) micro-CT; (**b**) feedstock (SEM) (100×); (**c**) feedstock (SEM) (500×); (**d**) feedstock (SEM) (1000×).

**Figure 9:**
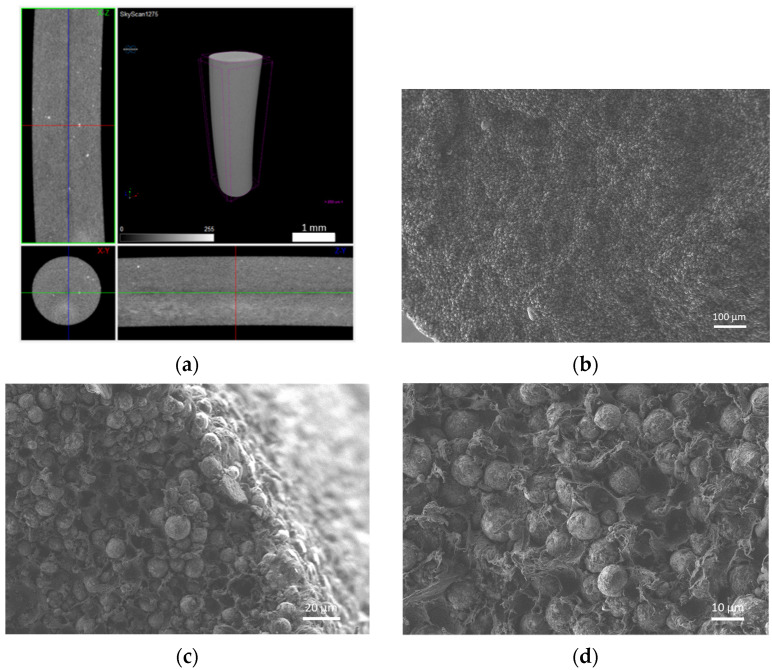
Filament B (green): (**a**) micro-CT; (**b**) feedstock (SEM) (100×); (**c**) feedstock (SEM) (500×); (**d**) feedstock (SEM) (1000×).

**Figure 10:**
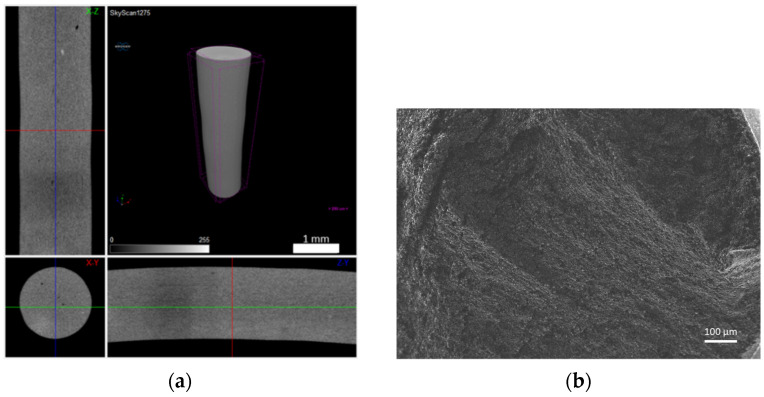

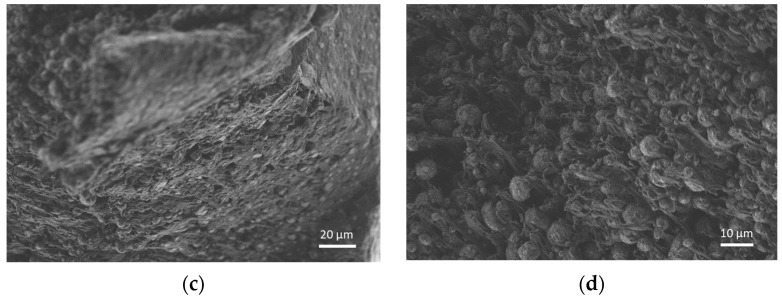
Filament C (green): (**a**) micro-CT; (**b**) feedstock (SEM) (100×); (**c**) feedstock (SEM) (500×); (**d**) feedstock (SEM) (1000×).

The filaments were analysed in terms of homogeneity (distribution of Cu powder particles into the polymeric material). Figure 8, Figure 9 and Figure 10 show micro-CT and SEM images of each type of filament with different particles size and particle size distributions. Filament A showed small pores distributed randomly throughout its volume. This could be the result of the large size distribution and particle size, which severely affects the powder behaviour during extrusion. Particle mobility is highly dependent on particle size. Filament B was almost an ideal case since there was a uniform distribution and low interparticular distance, with no discernible defects (within the micro-CT resolution). Filament C showed random pores, even though less prevalent and smaller than in filament A. SEM micrographs indicated apparent high mobility of the binder and additives. This low wettability can severely affect the sintering and debinding dynamics. Moreover, micro-CT volume rendering of the filaments revealed low-to-no ovality on the different filaments and a constant diameter.

**Figure 11:**
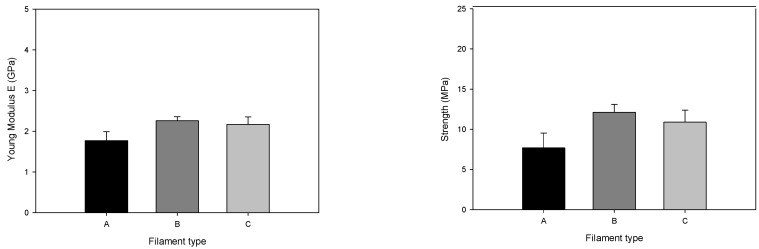

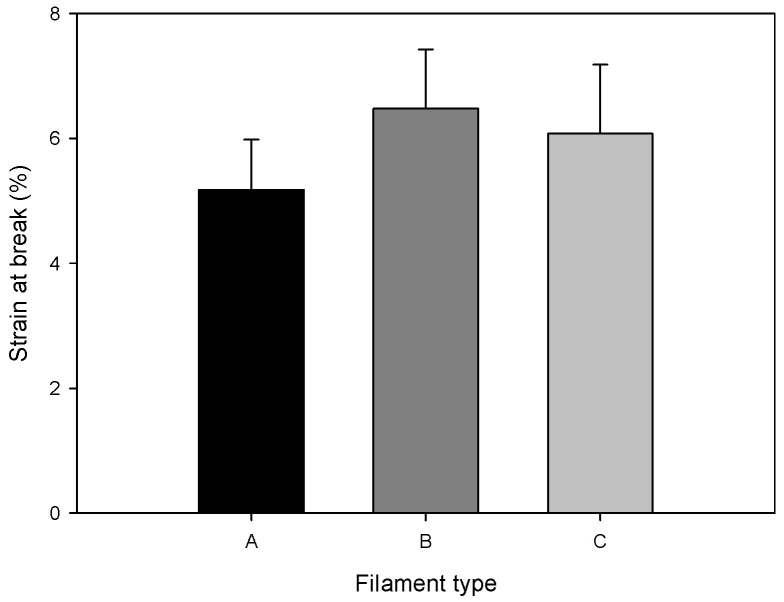
Young’s modulus, maximum tensile strength, and strain of different filaments (tensile tests).

**Figure 12:**
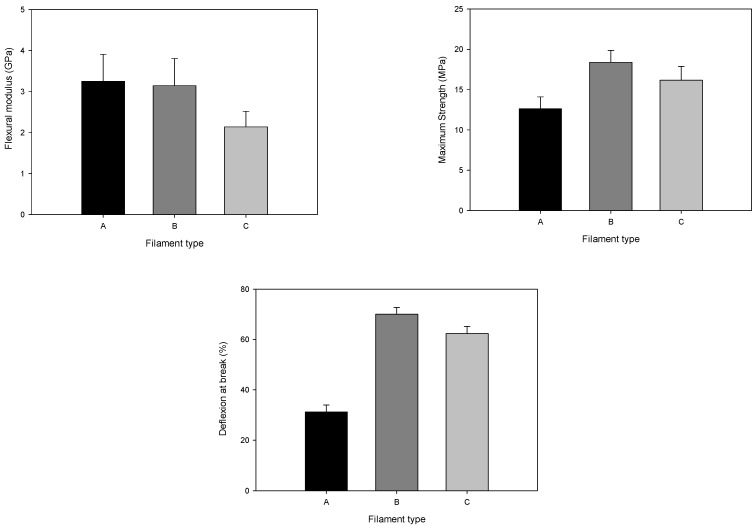
Flexural modulus, maximum strength, and deflection at break of different filaments (three-point bending test).

After detailed analyses of the filament morphology, tensile tests (Figure 11) and flexural tests (Figure 12) were performed. The mechanical characterisation revealed that filament A had more fragile behaviour than the others in the tensile tests, consistent with the porosity throughout the filament volume. Nevertheless, the tensile strength was the highest for filament B (12.1 MPa), followed by filament C (10.9 MPa), and the lowest value was measured for filament A (7.6 MPa). This decrease seems to be influenced by particle size and particle size distribution. Young’s modulus showed the highest value for filament B (2.2 GPa). The mechanical results support the detailed analysis made by micro-CT. Flexural tests revealed that the highest deflection was detected in the filament with the highest homogeneity and fewer defects, which is filament B. Even so, the behaviour of the flexural modulus was very similar for the three different filaments. The high deflection at break and low flexural modulus was already established to be printable by [29].

#### 4.2.2. Strands

**Figure 13:**
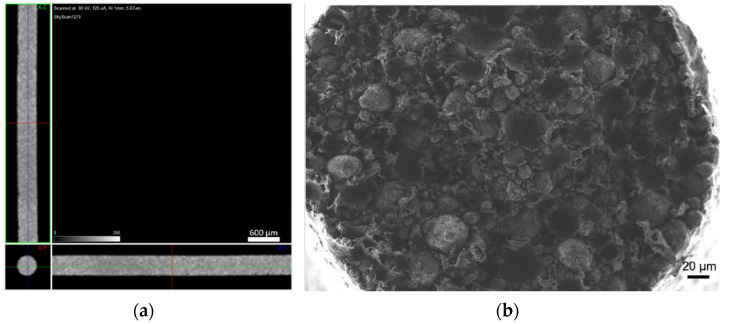
Strand A: (**a**) micro-CT and (**b**) cross section (SEM).

**Figure 14:**
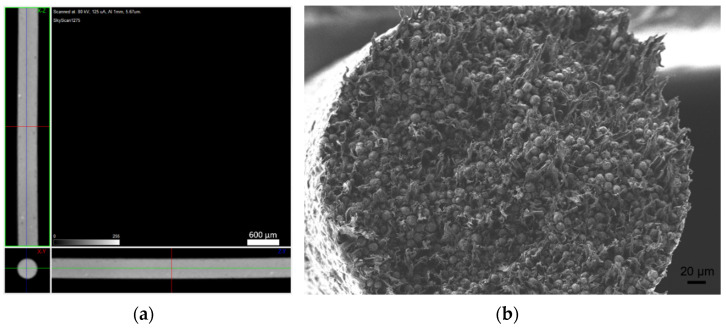
Strand B (**a**) micro-CT and (**b**) cross section (SEM).

**Figure 15:**
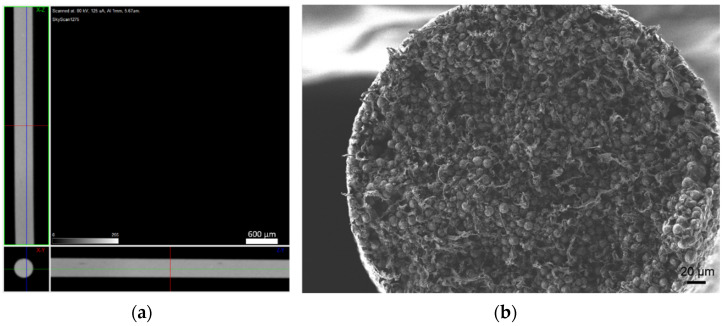
Strand C (**a**) micro-CT and (**b**) cross section (SEM).

The strands, subsequent to printer extrusion to build the 3D object, were analysed. The aim was to highlight the influence of the small nozzle (diameter = 400 μm) on powder distribution, which would be organised similarly in the final 3D object. The micro-CT analysis for the whole volume of the filament was confirmed using SEM. In the first case, powder A resulted in a non-uniform strand (denominated strand A), with larger particles randomly distributed throughout the volume (Figure 13). Henceforth, the strands were denominated by the powder name of their constituents. Micro-CT (high-density particles with a diameter larger than the lowest resolution, 5.67 µm, are in white) and the SEM images show particle distribution in the strand. In strand B, even though a low wettability of the binder + additives could be observed, had a very good distribution of the powder and interparticular distance, as seen in the original filament form (Figure 14). The results of strand C indicated that small defects, enlarged in the extrusion direction, may appear during printing and may be caused by the small hole diameter and particle compaction behaviour (Figure 15).

#### 4.2.3. The 3D Objects after Shaping

Hereafter, the different 3Dobjects resulting from strands A, B, and C were designated by A, B, and C, respectively.

**Figure 16:**
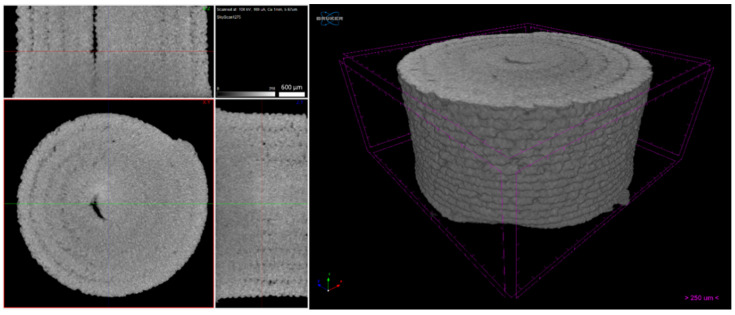
Three-dimensional object A (micro-CT).

**Figure 17:**
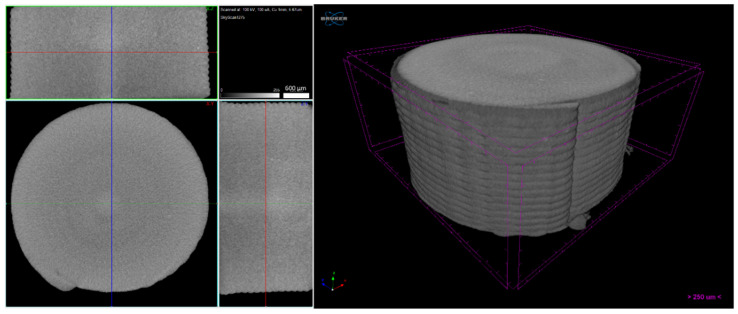
Three-dimensional object B (micro-CT).

**Figure 18:**
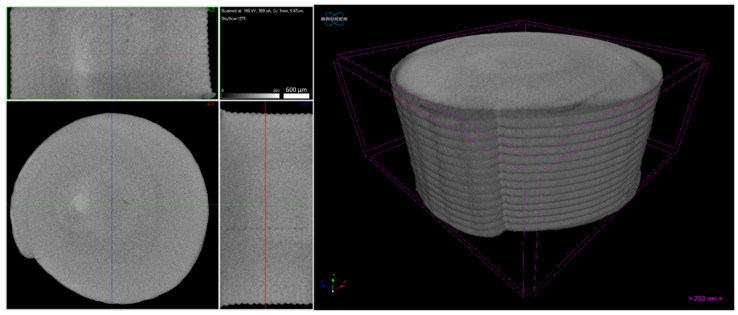
Three-dimensional object C (micro-CT).

Micro-CT of the green 3D objects (Figure 16, Figure 17 and Figure 18) was produced to observe the influence of powder size and powder distribution on the quality of a final 3D object produced with the same parameters. Since copper is a dense material, which affects the X-ray behaviour on micro-CT, as previously mentioned, only high-dimension pores were detected. Furthermore, 3D object A had a large number of defects that started halfway through building the object and a large open pore resulting from the use of the spiral printing strategy. This may be the result of the nozzle lacking heated bed pressure, which makes the strands flattened against the surface since the distance between the nozzle and substrate is not the same as each strand height. Similar to the filament, 3D object B seems to have no apparent defects and presents a better geometrical accuracy than A. object C also shows high density, but the micro-CT suggests that small defects may be present in its centre. This can be due to the extrusion multiplier parameter, as it may be creating enough strand flattening to mitigate the lack of the density apparent in each filament strand.

### 4.3. Debinding and Sintering

**Figure 19:**
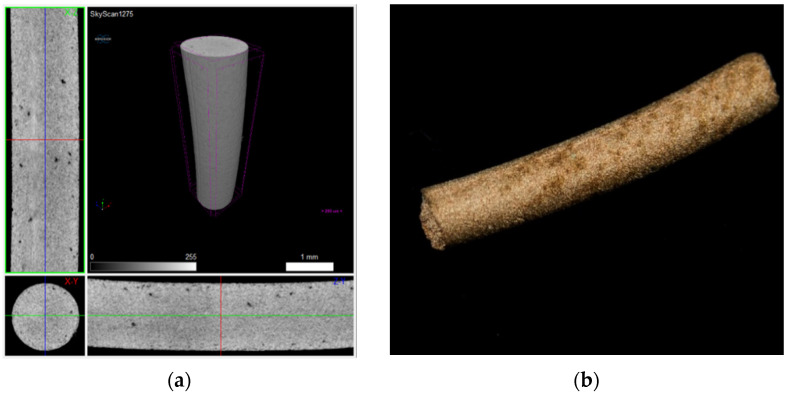
Filament A sintered: (**a**) micro-CT and (**b**) macrography.

**Figure 20:**
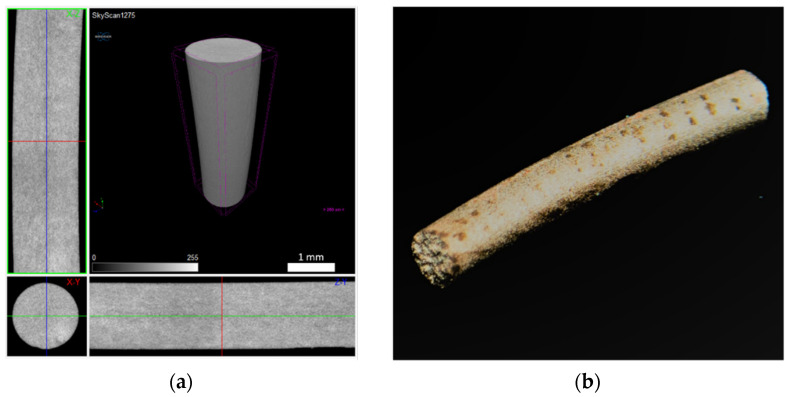
Filament B sintered: (**a**) micro-CT and (**b**) macrography.

**Figure 21:**
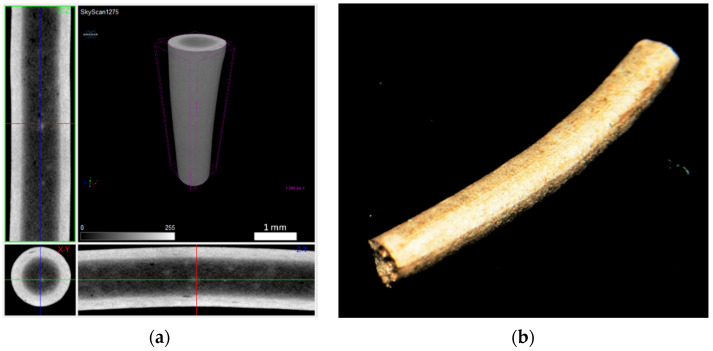
Filament C sintered: (**a**) micro-CT and (**b**) macrography.

These stages, transversal to the greens (filament and 3D object), were applied only to the filaments. To prove the copper feedstocks’ sinterability and avoid the influence of printing parameters on the final density, the three different filaments underwent the debinding (brown) process and were sintered. Figure 19, Figure 20 and Figure 21 show a micro-CT and a macrography of the sintered filaments. If A and B show the same defects, filament C had different behaviour. The external part of the filament was sintered, and the central part had a low density, which means it was not sintered. This can be attributed to copper oxide present in filament C, resulting in a barrier to the sintering of the central part. In fact, the high surface area of powder C was responsible for the highest oxidation.

### 4.4. Microstructures and Hardness

**Figure 22:**
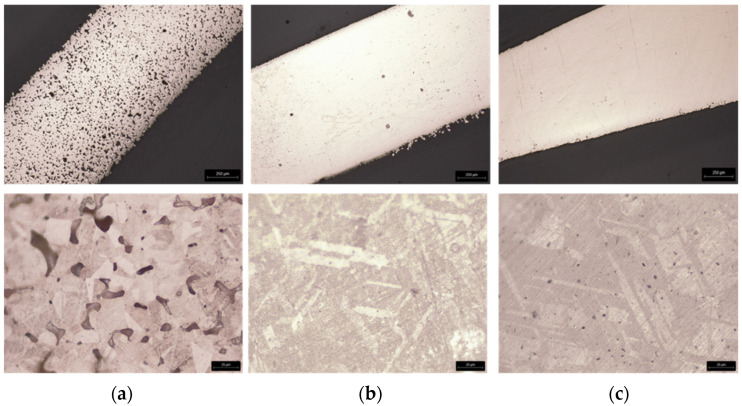
Macrography (top, 20×) and microstructure (OM) (bottom, 200×) of sintered filaments after etching: from powder A (**a**), B (**b**), and C (**c**).

Figure 22 shows different representative metallographic images of sintered copper MEX filaments. All the sintered filaments presented a typical microstructure of copper. However, it is clear that A and C presented a significant porosity, A with higher porosity than C.

In spite of C having the smallest particle size and particle size distribution, and consequently, the highest surface area, they had a difficult sintering process, evident in micro-CT. This behaviour is due to powder oxidation.

Table 5 summarises the different microhardness values measured in MEX 3D sintered objects from powder A, B, and C for the same thermal treatment (debinding and sintering) conditions. The microhardness values, particularly for powder C, support the presence of copper oxide ‘reinforcement’ on the powder surface [30,31].

## 5. Conclusions

Highly filled composite materials were prepared with 61 vol.% of copper with three different particle size and particle size distributions. It was concluded that the filaments were successfully extruded with a homogeneous distribution of powder, binder, and additives, particularly considering the absence of pressure effect during extrusion. Moreover, it became evident that the green filament based on powder B had the maximum strength and deflexion at break. After debinding and sintering, the best filament (B) had a D_50_ close to 11 µm with a monomodal particle size distribution, shape factor 1, and a surface similar to other powder. The only negative aspect of copper that must be solved in MEX was the powder oxidation resulting from powder preparation, which depends on particle size, and it became evident by the density values of copper powder. This negative aspect, which had consequences in sintering, must be overcome by the selection of another environmental atmosphere than Ar + H_2_, for example, H_2_ [32].

## Figures and Tables

**Table 1:** Composition of binder and additives.

	Master Binder	Additives	
		Backbone	Plasticiser
Vol (%)	77.5	17.5	5.0
Density (kg/m^3^)	970	1025	96.5

**Table 2:** Diameter of powder particles (D_10_, D_50_, D_90_) and their density.

Powder	D_10_ [µm]	D_50_ [µm]	D_90_ [μm]	ρ [Kg/m^3^]
A	8.57	28.00	46.60	8896
B	7.75	11.30	16.20	8648
C	1.95	3.97	6.67	8427

**Table 3:** Particle size (D_50_), particle size distribution, shape factor, and surface (topography and structure).

Powder	Particle Size D_50_ (μm)	Particle Size Distribution	Shape Factor *	Topography	Phase Composition
A	28.00	Bimodal	≈1	Uniform with some satellites	Cu + traces of copper oxide
B	11.30	Unimodal	≈1	Uniform with some satellites	Cu + copper oxide
C	3.97	Unimodal	≈1	Uniform with some satellites	Cu + copper oxide

* Shape factor close to 1 means spherical particle.

**Table 4:** CPVC of feedstocks, density, and maximum torque.

Feedstock	Cu (vol.%)	Binder + Additive (vol.%)	ρ (Kg/m^3^)	Torque (N·m)
A	61	39	5345	3.8
B	61	39	5330	5.1
C	61	39	5205	4.4

**Table 5:** Microhardness of the sintered filament.

Specimens	A	B	C
Microhardness (HV0.1) (10 measurements)	68 ± 8.9	65 ± 2.7	80 ± 2.3

## Data Availability

Data sharing is not applicable to this article.
